# Evidence for the role of transcription factors in the co-transcriptional regulation of intron retention

**DOI:** 10.1186/s13059-023-02885-1

**Published:** 2023-03-22

**Authors:** Fahad Ullah, Saira Jabeen, Maayan Salton, Anireddy S. N. Reddy, Asa Ben-Hur

**Affiliations:** 1 Department of Computer Science, Colorado State University, Fort Collins, CO, USA; 2 Biochemistry and Molecular Biology Department, The Hebrew University Faculty of Medicine, Jerusalem, Israel; 3 Department of Biology, Colorado State University, Fort Collins, CO, USA

**Keywords:** Alternative splicing, Intron retention, Deep learning

## Abstract

**Background:**

Alternative splicing is a widespread regulatory phenomenon that enables a single gene to produce multiple transcripts. Among the different types of alternative splicing, intron retention is one of the least explored despite its high prevalence in both plants and animals. The recent discovery that the majority of splicing is co-transcriptional has led to the finding that chromatin state affects alternative splicing. Therefore, it is plausible that transcription factors can regulate splicing outcomes.

**Results:**

We provide evidence for the hypothesis that transcription factors are involved in the regulation of intron retention by studying regions of open chromatin in retained and excised introns. Using deep learning models designed to distinguish between regions of open chromatin in retained introns and non-retained introns, we identified motifs enriched in IR events with significant hits to known human transcription factors. Our model predicts that the majority of transcription factors that affect intron retention come from the zinc finger family. We demonstrate the validity of these predictions using ChIP-seq data for multiple zinc finger transcription factors and find strong over-representation for their peaks in intron retention events.

**Conclusions:**

This work opens up opportunities for further studies that elucidate the mechanisms by which transcription factors affect intron retention and other forms of splicing.

**Availability:**

Source code available at https://github.com/fahadahaf/chromir

**Supplementary information:**

The online version contains supplementary material available at 10.1186/s13059-023-02885-1.

## Introduction

Alternative splicing is a widespread regulated phenomenon that enables a single gene to encode structurally and functionally different transcripts [1, 2]. The primary forms of alternative splicing are exon skipping, intron retention (IR), and alternative $3'$ and $5'$ splicing. While exon skipping is well studied, IR remains an under-appreciated phenomenon [3]. IR is the primary form of alternative splicing in plants [4, 5], and recent studies have shown it to have a high prevalence in human [6, 7]. Many disease-causing mutations are pathogenic through their effect on splicing, often leading to IR [6, 8, 9]. For example, IR is associated with genetic variants with deleterious effect on the function of tumor suppressor genes [10].

**Fig. 1:**
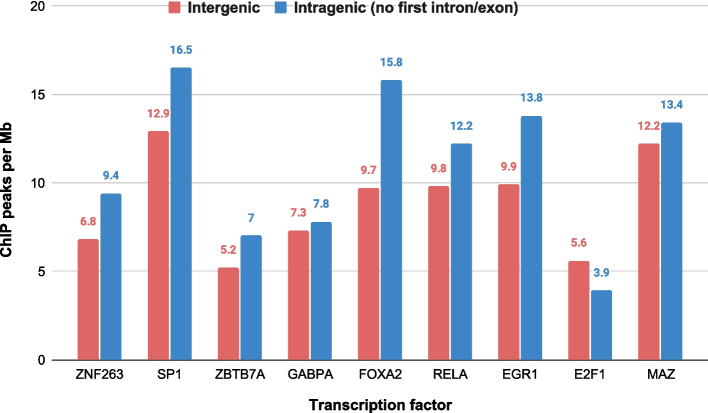
TF binding across the genome. We computed the number of ChIP-seq peaks per Mb for a selection of TFs, distinguishing between intergenic and intragenic peaks. Intergenic counts excluded the promoter region while intragenic counts excluded the first exon and intron to remove the effect of the promoter region

In recent years, efforts have been made to understand the regulation of IR and the factors that contribute to it. Braunschweig et al. [7] recently published a draft IR splicing code: a predictive model that uses a total of 136 features thought to be associated with IR in mammals. These features include base composition of an intron and its flanking exons, features that describe gene architecture, and splice site strength. This model is limited in that it does not model sequence elements that contribute to the regulation of IR. The discovery that splicing occurs co-transcriptionally suggests that chromatin state might be relevant to alternative splicing [11, 12]. Recent work provides evidence for the regulatory contribution of chromatin state to exon skipping [13], and our labs have provided preliminary evidence for its role in regulating IR in plants [14]. Open chromatin is one of the most important signatures for the study of chromatin structure. One of the primary tools for probing open chromatin is through exposure of DNA to deoxyribonuclease I (DNase I), which is an enzyme that cleaves DNA. Regions of the genome that are sensitive to its action—DNase I hypersensitive sites (DHSs)—have been used as an indicator of chromatin accessibility in vivo [15]. DHSs have been used extensively to identify several types of regulatory elements such as promoters, enhancers, silencers, and insulators [16, 17]. Furthermore, when a regulatory protein binds DNA, it protects it against the action of DNase I [18] and leaves a footprint which can be identified using DNase I-seq data [19, 20]. When it comes to alternative splicing, Mercer et al. [13] have shown an association between DHSs and exon-skipping, reporting that higher numbers of DHS-containing exons are alternatively spliced. Furthermore, this study reports that DHS exons with promoter and enhancer-like features have a higher fractional overlap with alternative splicing. Braunschweig et al. [7] explored the co-transcriptional regulation of splicing, reporting higher chromatin accessibility in retained introns and that polymerase II elongation speed affects IR and vice-versa. In another work, it has been reported that zinc finger transcription factors (TFs) have a regulatory role in exon skipping [21]. Recently, we studied the association between chromatin accessibility and intron retention in plants [14]. We identified potential regulatory elements occurring primarily in the 3′ flanking exons of IR events, several of which significantly match plant zinc finger binding site motifs. As further motivation for considering the role of TFs in splicing regulation, we provide evidence for extensive TF binding within human genes using ChIP-seq data. We collected ChIP-seq data in K562 for 11 different TFs and computed the number of peaks per Mb in intergenic regions and compared it to the number of peaks in intragenic regions. The results shown in Fig. 1 clearly demonstrate that for this selection of TFs the number of intragenic peaks is higher. A similar observation was made in plants [22]. This suggests a regulatory role of TFs beyond the regulation of gene expression.

Deep neural networks have become the tool of choice for exploring complex biological phenomena such as gene expression and chromatin state [23–28]. A remarkable advantage of these models is their ability to capture the underlying patterns in large noisy datasets directly from sequence with minimal pre-processing, learning motifs of the regulatory proteins involved as part of the training process. Deep learning has been used in genomics for TF binding prediction [29–31], chromatin accessibility analysis [23–25], prediction of chromatin structure and its modifications [32, 33], identification of RNA-binding protein sites [28, 34, 35], and prediction of splice site usage from sequence [36, 37]. Several labs have developed sophisticated models of exon skipping on the basis of large collections of genomic features [38–40], but have not considered the role of chromatin.

In this study, we demonstrate that deep learning models can distinguish with good accuracy regions of open chromatin associated with IR from other intronic regions of open chromatin. By analyzing the motifs learned by the network, we find that specific families of TFs are associated with IR events, mostly members of the zinc finger family of TFs; results of ChIP-seq experiments for multiple zinc finger TFs in the K562 cell line, one of three tier 1 ENCODE cell lines, support our findings for this association. Analysis of knockdown experiments of some of these TFs suggest they function as splicing enhancers by binding the flanking exons of IR events. Our work provides convincing evidence for a novel role of TFs in the regulation of IR, proposing a promising direction for further research.

## Results

### DHSs associated with IR can be accurately predicted from their sequences

**Fig. 2:**
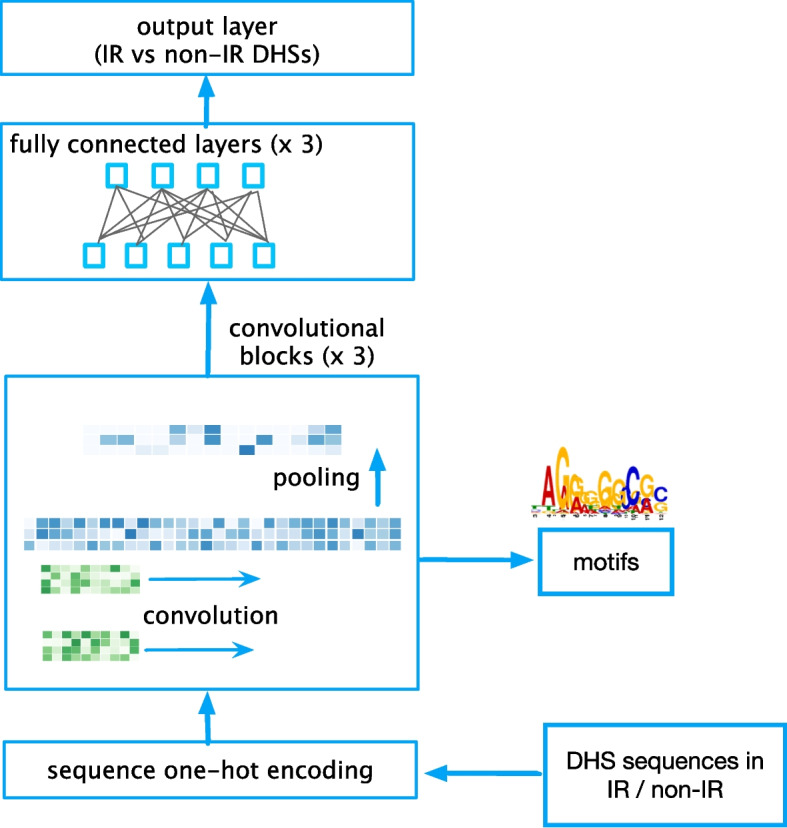
A deep learning model for predicting whether a region of open chromatin exhibits IR. The model receives as input the sequences of intragenic DHSs labeled as associated with IR or non-IR; the one hot encoding is processed through three layers of convolution, followed by three fully connected layers and the output layer that predicts a binary response that indicates whether a DHS exhibits IR or not. The convolutional filters of the first layer are used to extract position weight matrices (PWMs) that are searched against a database of known TFs

**Fig. 3:**
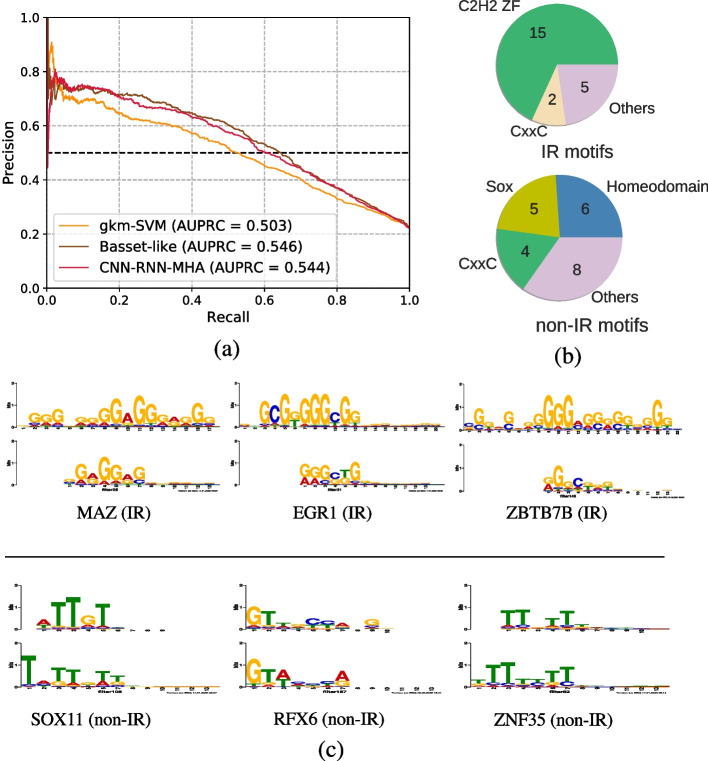
Classification accuracy and motifs detected by the network. **a** Precision-recall curves for the two deep learning architectures and the gkm-SVM. The AUC-PRC values are also provided in the legend. **b** The distribution of TF families enriched in IR vs non-IR events. **c** The top three matches for the IR and non-IR convolutional layer filters against the CISBP database. In each match, the known TF motif is shown in the top row and the bottom row shows the CNN filter/motif. The motifs shown above the line are associated with IR DHSs, and those below the line are associated with non-IR DHSs

In order to discover the sequence elements that regulate IR via its coupling with chromatin state, we trained and evaluated deep learning models to distinguish DHSs associated with IR events from non-IR DHSs in human and assessed and compared their performance. As IR DHSs, we used regions in which a DHS overlapping IR event was detected in at least one DNase I-seq experiment in a compendium of 164 samples; IR events were extracted from the Ensembl gene models as described in the “Methods” section. For non-IR DHSs, we used intronic regions exhibiting a DHS where no IR is known to occur. In this work, we chose to focus on the purely convolutional architecture shown in Fig. 2, that has demonstrated its effectiveness for predicting chromatin accessibility by Kelley et al. [23]. The model hyperparameters were tuned for our problem as described in the “Methods” section. Using this model we obtained accuracy of 0.546 as measured using the area under the precision-recall curve (AUC-PRC) (see Fig. 3a). A more sophisticated model that uses a combination of convolutional and recurrent layers with multi-head attention achieved a similar level of accuracy (see Fig. 3a and Additional file 1: Fig. S1). We note that both deep learning architectures outperformed a baseline approach that uses the gkm-SVM method [41]. This method achieved an AUC-PRC of 0.503. ROC curves are provided in Additional file 1: Fig. S1.

Our results were generated using a one-hot encoding of the sequence of DHS regions. We note that word2vec embeddings provided a small improvement in accuracy, as shown in Additional file 1: Fig. S1. However, this came at a cost of reduced interpretability of the models, leading to reduced ability to infer motifs associated with the learned convolutional filters (see discussion in the Additional file 1). Therefore, we chose to focus on models that used one-hot encoding as input. During the revision of the manuscript, we discovered 72 duplicate DHSs out of the 7500 training examples in the original dataset provided by Kelley et al. [23]. Some of these occurred across the train and test set. At a threshold of 80% sequence identity, we also found using CD-HIT [42] 39 sequences whose similarity is above that threshold. We re-trained the classifier without the duplicates and similar sequences and found that the accuracy was unchanged.

### The zinc finger family of TFs are enriched in IR events

The filters of convolutional networks can be readily interpreted as motifs. To do so, we implemented the strategy described elsewhere [23, 29] (see “Methods” section for details). We analyzed the motifs that were derived from the convolutional filters for both the top positive and the top negative examples and searched both sets of motifs against the Human CIS-BP TF database [43] using TomTom [44]. We found that 22 IR-associated motifs had significant hits against multiple known human TFs at a *q*-value $< 0.01$. In comparison, 23 of the non-IR motifs had significant matches. Figure 3c shows some of the top hits for both IR and non-IR motifs, and a complete list is found in the github repository of the project. The median information content of the IR motifs was 4.21, and 4.26 for the non-IR motifs. The other architectures provided motifs with similar information content (see Additional file 1: Tables S4 and S5). Furthermore,when comparing the TF hits for the IR motifs with an adjusted *p*-value of 0.01 or better for the three different architectures (the purely convolutional network and variants that include attention with and without a recurrent layer), we found that 21 out of the 25 motif hits discovered by the purely convolutional architecture were common across the three architectures.

Most of the IR motifs had significant hits in the C2H2 zinc finger family of TFs (C2H2 ZF). Non-IR motifs on the other hand, were predominantly matched to the Homeodomain and Sox families of TFs (see Fig. 3b). Zinc finger TFs have previously been implicated in the regulation of alternative splicing [21], particularly exon skipping. Here we report a role of this family in the regulation of IR. We note that some of our filters do not match a unique transcription factor. For example, the filter that matched MAZ, was also a good match for ZNF263. This is not surprising due to the similarity of the binding sites of zinc finger TFs. Below we provide additional evidence for the role of zinc finger TFs in regulating IR.

We also searched for motif matches to RNA-binding proteins in the CISBP-RNA database [45] using the same methodology employed for TFs. At the same *p*-value threshold used for searching for TF hits, we found three significant hits in the IR motifs, and two significant hits in non-IR motifs. This is compared with 22 TF hits in IR motifs and 23 TF hits in non-IR motifs. Details of those matches are found in the github repository of this project. This suggests that TFs play a major role in IR in comparison to RNA-binding proteins, perhaps as a result of our focus on regions of open chromatin.

### TF ChIP-seq analysis supports model predictions

To validate our findings using experimental data, we downloaded K562 ENCODE ChIP-seq datasets for all the zinc finger TFs identified by our model, resulting in six datasets. Using these datasets, we tested TF binding enrichment in IR vs. non-IR events, following a strategy similar to our previous work [14]: for each TF, we measured the overlap of its ChIP-seq peaks with IR and non-IR events and tested its significance using the Fisher exact test. All the TFs demonstrated highly significant enrichment in IR events (see Table 1), validating our in silico findings that the C2H2 ZF family plays a role in the regulation of IR.

**Table 1:** Enrichment of C2H2 ZF TF binding in IR compared to non-IR events quantified using ChIP-seq peaks of the corresponding TF. We note that for the bottom three TFs, the *p*-value is for the significance of enrichment in non-IR events

TF	IR TF occupancy (%)	Non-IR TF occupancy (%)	*p*-value
EGR1	12.51	7.16	1.27E−45
MAZ	11.42	6.06	9.75E−51
ZBTB7A	10.6	5.52	3.15E−49
SP1	3.04	1.53	7.64E−16
SP2	1.32	0.77	7.21E−06
ZNF263	1.14	0.67	2.81E−05
FOXK2	5.2	6.12	3.01E−04
GATA1	0.77	1.07	5.0E−03
JUN	2.68	4.86	3.21E−23

### RNA-seq of TF Knockdowns suggest IR TFs function as splicing enhancers

To obtain a better understanding of the way the TFs predicted to be associated with IR function to regulate IR, we analyzed RNA-seq datasets of the K562 cell line with knockdown/silencing of several IR-associated TFs: MAZ, SP1, SP2, and E2F4. To evaluate the effect of the knockdown of each TF, we looked for differential IR events in the knockdown samples with respect to baseline K562 using iDiffIR [46]. In all cases, there were many more up-regulated IR events, i.e., events with increased IR with respect to the wild-type that are statistically significant at a *p*-value of 0.05 and above (see Table 2). This suggests that these TFs predominantly function as splicing enhancers. Furthermore, we searched for the hits for the motifs of each TF in the differentially retained introns compared to introns that are not differentially retained. We found that in introns that showed a statistically significant increase in IR levels, there was a much higher number of hits for the motif of each TF compared to IR events where no significant difference in retention was observed; this difference was statistically significant (see Table 2), further support for the role of these TFs as splicing enhancers.

**Table 2:** RNA-seq results for TF knockdown experiments in K562. For each TF, we provide the number of statistically significant IR events that are up-regulated (down-regulated), i.e., exhibit increased (decreased) retention with respect to the wild-type. Within the up-regulated events we provide the number of events with occurrences of the motif compared to the background set composed of IR events. This is done in both the intron and the flanking exons. The significance of the difference in the rates of occurrence of the motif is provided in the last column. In all cases the significance was exhibited in the flanking exons, except for E2F4 which exhibited similar levels of significance in both the introns and flanking exons

TF	Up-regulated	Down-regulated	Motif occurrences		*p*-value
			Intron	Exon	
MAZ	69	34	23% (25%)	31% (16%)	0.001
SP1	86	18	69% (64%)	86% (51%)	1.3E−9
SP2	99	25	31% (27%)	41% (16%)	1.4E−9
E2F4	174	58	18% (12%)	18% (11%)	0.008

### Regulatory interactions between TFs in IR events

**Fig. 4:**
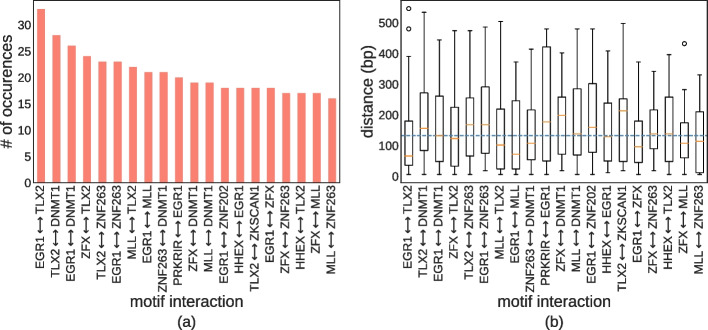
TF interactions. **a** The most frequent TF interactions in IR events. **b** The distribution of distances between detected TF interactions. The dotted blue line represents the median distance across all significant interactions

It is well known that TFs often function in tandem with each other to regulate their targets. To extract such regulatory interactions, we have recently developed a method called SATORI to interpret deep architectures that use *attention* layers and extract statistically significant interactions between its convolutional filters [47]. SATORI uses the so-called attention matrix, which encodes relations between different positions of the sequence; subsequent analysis of the convolutional filters that are active provides a profile of interactions between pairs of TFs that are associated with those filters. By comparing those profiles to those in a background set of sequences, we obtain interactions that are statistically significant. Using SATORI, with the negative examples as a background set to assess statistical significance we detected over 400 TF interactions in DHSs associated with IR at a significance level of 0.05. The top 20 predictions are shown in Fig. 4, and the complete list is provided in the results directory of the project’s github repository. A histogram for the number of interactions between TF families is provided in Additional file 1: Fig. S2. A majority of the interactions involve the C2H2 ZF family, which is expected since C2H2 ZF TFs have the most hits from our model. To validate these interactions, we searched for matches in annotated interactions in the TRRUSTv2 [48] database that annotates TF regulatory roles and their interactions by text-mining the biomedical literature. Of the interactions detected by our model, we found 23 overlapping interactions in TRRUSTv2, which currently contains 8324 interactions. This is highly significant, with a *p*-value equal to 0 in a hypergeometric test. We also obtained significant overlap with protein-protein interactions from the HIPPIE database [49]: 17 of the detected interactions had support in HIPPIE, with a hypergeometric *p*-value of 1E−52. The interactions overlapping with TRRUSTv2 and HIPPIE database are listed in Additional file 1: Tables S2 and S3, respectively. As further support for predicted interactions, we looked at ChIP-seq data in K562 for the interactions EGR1-MAZ and EGR1-ZNF263 and evaluated the overlap between the peaks in intragenic regions. For the EGR1-MAZ interaction, we found 21,592 intragenic peaks for EGR1 and 16,613 for MAZ. Out of those peaks, 9065 were within 150 bp of each other. Using Locus Overlap Analysis [50, 51] to evaluate the significance of the overlap, we obtained a *p*-value of 0. For the EGR1-ZNF263 interaction we found 21,592 intragenic ChIP-seq peaks for EGR1 and 1619 for ZNF263. Out of those peaks, 715 were within a window of 150 bp of each other with a *p*-value of 1E-17. Finally, we looked at the average distance between motifs predicted to interact and found that TF motifs preferentially interact in proximity, with a median distance of 120 bp, which is significantly less than what we would expect by chance (*p*-value of 3.65E−13 in the Mann-Whitney *U* test). These results suggest that regulation of IR is orchestrated by complex interactions among TFs, predominantly from the C2H2 ZF family.

## Discussion

In our motif analysis, we found that the C2H2 zinc finger family of TFs has a strong association with IR events: Over $65\%$ of the motifs associated with IR have significant hits to C2H2 ZF TFs. This is consistent with previous work reporting that zinc finger TFs influence exon skipping [21], and suggests that the C2H2 ZF family plays an important role in the regulation of alternative splicing in general.

To validate our predictions on the association of these TFs with IR, we used ChIP-seq data for multiple zinc finger TFs: MAZ, EGR1, SP1, ZBTB7A, SP2, and ZNF263. We observed much higher occupancy of these TFs in IR events in the K562 human cell line, validating the model’s predictions. Robson et al. [52] have reported that MAZ4 elements that contain four copies of the MAZ binding sequence influence alternative splicing. More recently, it was demonstrated that MAZ acts in conjunction with CTCF to remodel chromatin to affect changes in alternative splicing [53]. They have also demonstrated that like CTCF, MAZ can slow the elongation of RNAPII and affect splicing outcome.

There are multiple potential mechanisms by which TFs can affect co-transcriptional splicing. First, TFs are known to be critical in establishing chromatin state, which in turn can regulate alternative splicing by a purely kinetic model of the coupling between transcription and splicing whereby higher speeds of transcription in regions of accessible chromatin give less time for the spliceosomal machinery to recognize and splice those introns co-transcriptionally [11, 54, 55]. An alternative explanation of this phenomenon is that accessible chromatin is a mark of binding of TFs or other regulatory proteins that recruit splicing factors directly or indirectly through chromatin modifications to affect the outcome of splicing [7]. Wet-lab experiments are required to explore these hypotheses and provide more mechanistic details on how TFs regulate IR and other forms of alternative splicing.

Our model of retained introns considered only chromatin accessibility. There are other aspects of chromatin organization that can be considered: histone modifications and DNA methylation. Through their effect on chromatin organization, histone modifications impact the speed of RNAPII elongation and thereby alternative splicing [54]. Luco et al. [56] proposed the *adaptor system* model whereby DNA-binding proteins recognize a histone modification and recruit a splicing regulator that affects the splicing outcome (see also [57]). Methylation-dependent alternative splicing has been shown to be widespread [58], and its patterns have been observed to delineate exons and their boundaries [59, 60]. Histone modifications and methylation patterns can thus provide another layer of information relevant to the regulation of IR.

In this work, we focused on the local coupling of accessible chromatin and IR. We expect that non-local interactions through chromatin loop anchors like those that allow enhancers to affect promoter activity [61] can affect IR; evidence for their impact on exon skipping has recently been reported in human [62]. Recent work has demonstrated the role of a specific enhancer within a chromatin loop and its role in regulating alternative splicing [63]. Future work can incorporate them in the context of a comprehensive model of alternative splicing.

## Conclusions and future work

Using deep learning to model intragenic DHSs allowed us to explore the regulatory elements that are predictive of IR in an unbiased fashion and identify TFs as key contributors to the regulation of IR. Further experimental work is required in order to validate the role of TFs in IR regulation. This will be supported by extensions of the model that allow tissue-specific prediction of the IR state of regions of open chromatin, and create the chromatin-mediated IR code. Furthermore, the modularity of deep learning will allow the extension of the model to incorporate other sources of data indicative of chromatin state such as histone modifications. Much in the same way chromatin loop anchors allow enhancers to affect the activity of promoter regions and affect gene expression [61], there is recent evidence for their impact on exon skipping [62]. Therefore, we expect that chromatin interaction information captured by Hi-C or Micro-C data is likely to improve the model and provide a more holistic view of IR regulation. Such data can be incorporated in a deep learning model with modules that use graph convolution; recent work has shown the effectiveness of this approach for modeling various aspects of chromatin state [64].

## Methods

### Data collection, processing, and representation

We used DNase I-seq data from 125 human immortalized cell-lines and tissues from the ENCODE database [65] and 39 cell types from the Roadmap Epigenetics consortium [66] as processed by [23]: every DNase I-seq peak was extended to a length of 600 bp around its midpoint and adjacent peaks are greedily merged until no two peaks overlap by more than 200 bp. For our analysis we focused on over a million DHSs that occur within genes.

Next, we extracted IR events from the Ensembl GRCh37 (hg19) reference annotations, utilizing code from SpliceGrapher [67] and iDiffIR [46]. In total, we identified 58,305 unique IR events out of which, 15,400 had overlapping DHSs. These constitute our positive examples. We used a strict criterion requiring a DHS to overlap the retained intron, i.e., DHSs overlapping only the flanking exons did not qualify. All other intragenic DHSs that did not overlap an IR event were labeled as negative examples. The number of negative examples was roughly twice the size of the positive set.

We used two methods to transform the sequences into input for our neural networks: one-hot encoding and sequence embedding. For one-hot encoding, a sequence is represented as a $4\times N$ matrix where *N* is the length of the sequence. Each position in the sequence is represented by the columns of the matrix with a non-zero value at a position corresponding to one of the four DNA nucleotides. To represent a sequence using embedding, we first decomposed it into overlapping *k*-mers of length *k* and then used a word2vec model [68] to map each *k*-mer into an *m*-dimensional vector space. This gave us an embedding matrix of dimensions $(N-k+1)\times m$. This representation is designed to preserve the context of the *k*-mers by producing similar embedding vectors for *k*-mers that tend to co-occur.

### Network architecture

In this work, we investigated several network architectures. The primary network element, a one-dimensional convolutional layer, scans a set of filters against the matrix representing the input sequence. Formally, we can express the convolution operation as:

1 $$\begin{aligned} x_{i,j} = \sum _{a=0}^{A-1}\sum _{b=0}^{B-1}\varvec{W}_{a,b}^j \varvec{X}_{i+a,b}, \end{aligned}$$

where $\varvec{X}$ is the input matrix, *i* is the current output index, and *j* is the index of the filter. $\varvec{W}$ is the weight matrix with size $A\times B$ where *A* is the length of the filter (window size) and *B* is the number of input channels: 4 for DNA one-hot encoding, *d* in case of word2vec embeddings, and *number of previous layer filters* in case of higher convolutional layers. The output of a convolutional layer is produced by applying a non-linear activation function to the result of the convolution operation. We use the rectified linear unit (ReLU) which is given by:

2 $$\begin{aligned} f(x) = max(0,x) . \end{aligned}$$

Next, the size of the output is reduced by max-pooling where the maximum value in a window of a pre-determined size is selected. This reduces the input size for the next layer and also leads to invariance to small shifts in the input sequence.

We also incorporated a multi-head self-attention layer as the basis for an alternative deep learning model. Attention is a powerful feature that is able to model dependencies within an input sequence regardless of their distances [69]. By doing so, it guides the network to focus on relevant features within the input and ignore irrelevant information. Our implementation uses the same architecture used for the SATORI method [47], and consists of a single convolutional layer followed by a max-pooling layer and a multi-head attention layer. We also used a recurrent layer as an option in conjunction with the multi-head attention layer, since it provided improved performance in other datasets [47]. RNNs have an internal state that enables them to capture distant feature interactions in the input sequence. Specifically, we employed a bi-directional RNN with Long Short-Term Memory (LSTM) units [70]. In a bi-directional RNN, a forward and a backward layer are used that traverse the input in both directions, improving the model’s performance. The bi-directional LSTM layer was used between the convolutional layer and the multi-head attention layer. Code for all the architectures is available in the project’s github repository.

### Network training and evaluation

First, the data was split into training, validation, and test sets with $80\%$, $10\%$, and $10\%$ of the total data, respectively. Next, using the training and validation sets, we tuned the network hyperparameters by employing a semi-randomized grid search that uses a 5-fold cross-validation strategy. For the Basset-like model variant, we started with the hyperparameters reported in [23] and fine-tuned their values. The hyperparameters are summarized in Additional file 1: Table S1. All the models were evaluated using the test set using the area under the ROC curve (AUC-ROC) and the area under the precision-recall curve (AUC-PRC).

### Gapped kmer SVM

As a baseline we used the large-scale gapped kmer SVM (gkm-SVM), called the LS-GKM [41]. This version can handle bigger datasets (50k–100k examples) and exhibits better scalability. We run the package with the following parameters: $-m$ 20000 and $-T$ 16 which specify the size of the memory cache in MB and number of processing threads, respectively.

### Motif extraction and analysis

To interpret the deep learning models, we extracted sequence motifs using the weights (filters) of the first convolutional layer, similar to the methodology described by Kelley et al. [23]. We selected the positive examples (DHSs overlapping IR events) in the test set with prediction probability greater than 0.65. This cutoff was chosen as a trade-off between the number of qualified examples and confidence in the prediction. For the negative examples, we used a cutoff value of less than 0.35. Next, for each filter, we identified regions in the set of sequences that activated the filter with a value greater than half of the filter’s maximum score over all sequences. The highest scoring regions from all the sequences are stacked and for each filter, a position weight matrix is calculated using the nucleotide frequency and background distribution. We generated the sequence logos using the WebLogo tool [71]. The resulting PWMs were searched against the human CIS-BP database [43] using TomTom [44] with distance metric set to Euclidean. This was performed separately for motif hits in IR and in non-IR events, allowing us to associate motifs with IR or non-IR. For filters which yielded significant hits in both IR and non-IR, we chose the more significant hit.

### TF ChIP-seq analysis

We downloaded ChIP peaks of all the TFs that were detected as enriched in IR events from the ENCODE database [65]. Next, we used our previously published pipeline [14] to test the enrichment of a given TF ChIP peaks in IR events. Briefly, we quantified the overlap of ChIP peaks with IR events and compared them to the overlap with non-IR events. The significance of overlap was tested using the Fisher exact test. The accession numbers for the ENCODE K562 ChIP-seq datasets used in our analysis are as follows: EGR1: wgEncodeEH001646, MAZ: wgEncodeEH002862, ZBTB7A: wgEncodeEH001620, SP1: wgEncodeEH001578, SP2: wgEncodeEH001653, ZNF263: wgEncodeEH000630, FOXK2: GSE91647, GATA1: wgEncodeEH000638, JUN: wgEncodeEH000620.

### TF knockdown RNA-seq analysis

We downloaded RNA-seq data for knockdown of the following TFs in K562 from the ENCODE database: MAZ, SP1, SP2, E2F4 (accession numbers GEO:GSE88056, GEO:GSE127134, GEO:GSE127145, and GEO:GSE88612). In addition, we used wild-type K562 (accession number GEO:GSE33480). We computed differential IR events in the knockdown samples with respect to baseline K562 using iDiffIR [46]. Analysis of motif hits in differentially retained introns was performed using BioPython [72] using the motif of each TF retrieved from Jaspar [73].

### Discovering interactions between TFs

To discover regulatory interactions between TFs we used SATORI [47], which takes advantage of the self-attention matrix to infer possible interactions between sequence motifs. When running SATORI, we used the default parameters with exception to the following: --attncutoff 0.08 and --usevalidtest True. The postprocessing was performed using Jupyter notebooks provided with SATORI.

## Supplementary information

**Additional file 1.** Supplementary Material. The Supplement includes additional tables and figures.

**Additional file 2.** Review History.

## Data Availability

Code, training data, and additional results are available through the project’s github repository [74]. A copy of the repository is available through zenodo [75].
