# Supplementary material for: Evidence for the role of transcription factors in the co-transcriptional regulation of intron retention
Source: Genome Biol. 2023 Mar 22;24:53. doi: 10.1186/s13059-023-02885-1 (PMC10031921; doi:10.1186/s13059-023-02885-1)
Supplement: Supplementary file 1 — Additional file 1. Supplementary Material. The Supplement includes additional tables and figures. [file 13059_2023_2885_MOESM1_ESM.pdf]

Supplementary Material

Evidence for the role of transcription factors in the co-transcriptional regulation of intron retention

Fahad Ullah, Saira Jabeen, Maayan Salton, Anireddy SN Reddy, and Asa Ben-Hur

Embeddings and network interpretability

We used the Basset-like network (see Figure 2 in the main text) to demonstrate that embeddings reduce network interpretability. We used this model with both one-hot and word2vec representations of the input sequences. Interestingly, the average information content (IF) of enriched motifs significantly varied with the two input representations. When using the regular one-hot encoding, we find the motifs to be more informative and useful (mean IF = 4.0). The same is not true for word2vec embeddings where we get motifs with far lower information content (mean IF = 1.8).

Supplementary Figures

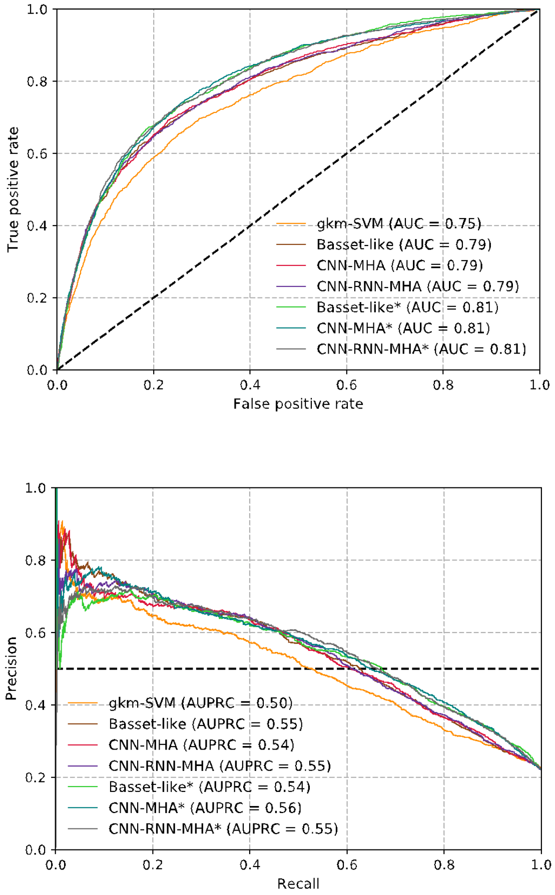

Figure S1: ROC (top) and Precision-Recall (bottom) curves for the different deep learning architectures as well as the gkm-SVM. Basset-like refers to a purely convolutional architecture similar to the Kelley et al.'s Basset method [23]; CNN-MHA uses a convolutional layer and multi-head attention; CNN-RNN-MHA uses a convolutional layer followed by an LSTM layer and a multi-head attention layer. Network architectures that use *k*-mer embeddings instead of one-hot encoding are indicated by an asterisk (*). The AUC and AUPRC values are provided in the legend.

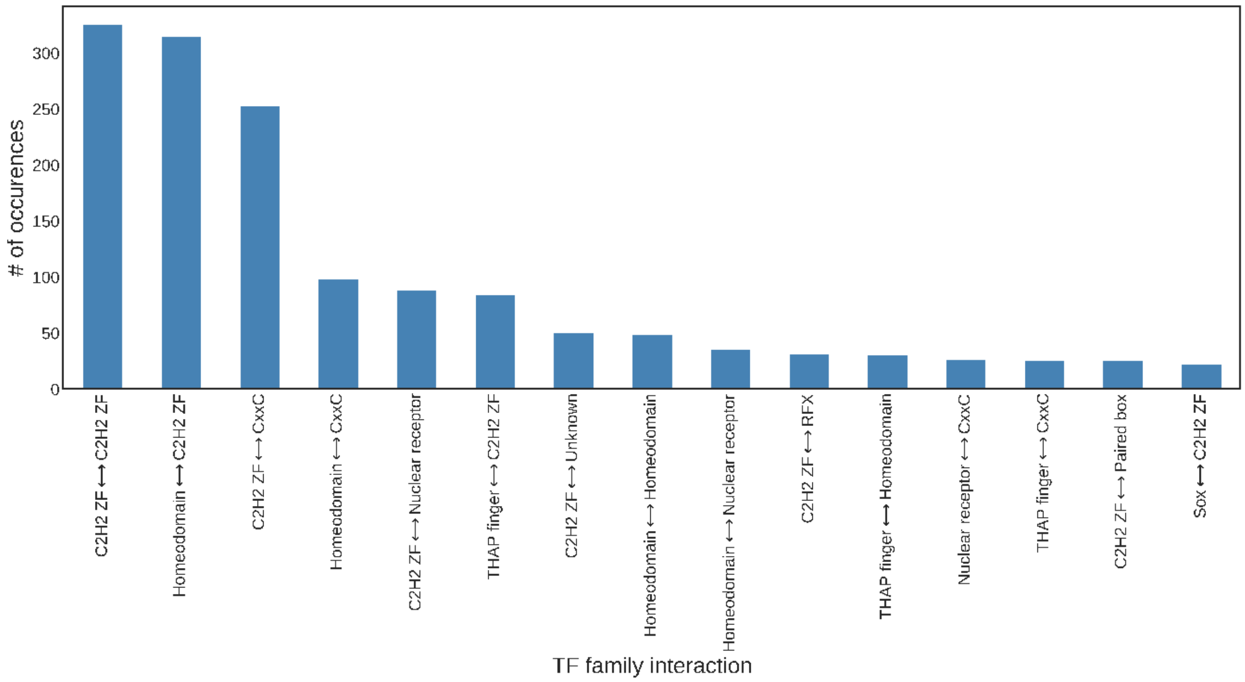

Figure S2: The most frequent interacting transcription factor families in intron retention events.

Supplementary Tables

Table S1: List of network hyperparameters.

Hyperparameter	Type	Description
singlehead_size	int	Size of the attention single head [default: 32]
num_heads	int	Number of heads in multi-head self-attention layer [default: 8]
multihead_size	int	Output size of the multi-head after concatenation [default: 100]
batch_size	int	Batch size in training/testing the model [default: 172]
use_RNN	bool	Choose whether to use the RNN layer. [default: based on model variant]
RNN_hidden_size	int	Size of the RNN layer. [default: 100]
CNN_filters	int	Number of CNN filters to use. [default: 200]
CNN_filter_size	int	Size of each CNN filter. [default: 13]
use_CNN_pool	bool	Use max pooling in the CNN layer. [default: True]
CNN_pool_size	int	Size of the max pooling window in CNN layer. [default: 6]
input_channels	int	Number of input channels. [default: 4 (for DNA sequences)]
num_epochs	int	Number of training epochs. [default: 30]
readout_strategy	string	Either to normalize the MHA output or flatten it. [default: "normalize"]
use_embd	bool	Whether to use the word2vec embeddings instead of 1-hot input [default: False]
embd_size	int	Size of the word2vec embedding vectors [default: 50]
embd_window	int	Size of the word2vec embedding window [default: 5]

embd_kmer	int	Length of the *k*mer (for word2vec embeddings) [default: 3]

Table S2: A list of TF interactions in IR events confirmed by the TRRUSTv2 database [48]. The level of significance(adjusted p-value) assigned by SATORI to each interaction is provided in the adusted_pval column.

TF_Interaction	TF1_Family	TF2_Family	adjusted_pval	frequency
DNMT1↔HINFP	CxxC	C2H2 ZF	2.39E-49	5
EGR1↔DNMT1	C2H2 ZF	CxxC	3.68E-45	26
ESR1↔DNMT1	Nuclear receptor	CxxC	5.58E-45	5
PAX8↔DNMT1	Paired box	CxxC	4.63E-42	4
ESR1↔PURA	Nuclear receptor	Unknown	5.14E-36	2
DNMT1↔ZBTB7A	CxxC	C2H2 ZF	1.49E-33	5
EGR1↔THRB	C2H2 ZF	Nuclear receptor	7.77E-33	9
DNMT1↔SP4	C2H2 ZF	CxxC	5.54E-30	4
ESR1↔PAX8	Nuclear receptor	Paired box	1.48E-27	1
HHEX↔PAX8	Paired box	Homeodomain	3.69E-22	4
E2F4↔DNMT1	E2F	CxxC	1.29E-19	1
ESR1↔EGR1	Nuclear receptor	C2H2 ZF	8.77E-15	6
ESR1↔RARG	Nuclear receptor	Nuclear receptor	1.03E-14	1
ESR1↔E2F4	Nuclear receptor	E2F	1.61E-14	1
EGR1↔PAX8	Paired box	C2H2 ZF	5.77E-13	5
EGR1↔PURA	Unknown	C2H2 ZF	8.00E-13	9
ESR1↔ZBTB7A	Nuclear receptor	C2H2 ZF	3.01E-07	1
ESR1↔SP4	Nuclear receptor	C2H2 ZF	1.26E-06	1
PURA↔SP4	Unknown	C2H2 ZF	3.50E-06	1
EGR1↔E2F4	E2F	C2H2 ZF	1.08E-05	2
EGR1↔SP4	C2H2 ZF	C2H2 ZF	2.36E-03	3

Table S3: A list of TF interactions in the IR events confirmed by the HIPPIE database [49]. The level of significance (adjusted p-value) assigned by SATORI to each interaction is provided in adjusted_pval column.

TF_Interaction	TF1_Family	TF2_Family	adjusted_pval	frequency
ESR1⟷IRX4	Nuclear receptor	Homeodomain	5.95E-65	1
ESR1⟷DNMT1	Nuclear receptor	CxxC	5.58E-45	5
HHEX⟷TLX2	Homeodomain	Homeodomain	4.43E-36	17
ESR1⟷PURA	Nuclear receptor	Unknown	5.14E-36	2
ESR1⟷ZHX1	Nuclear receptor	Homeodomain	8.48E-31	1
SOX1⟷ESR1	Sox	Nuclear receptor	3.88E-28	2
ESR1⟷PAX8	Nuclear receptor	Paired box	1.48E-27	1
ESR1⟷HINFP	Nuclear receptor	C2H2 ZF	1.41E-23	1
ESR1⟷SP7	Nuclear receptor	C2H2 ZF	3.41E-17	3
ESR1⟷EGR1	Nuclear receptor	C2H2 ZF	8.77E-15	6
ESR1⟷RARG	Nuclear receptor	Nuclear receptor	1.03E-14	1
ESR1⟷E2F4	Nuclear receptor	E2F	1.61E-14	1
EGR1⟷HINFP	C2H2 ZF	C2H2 ZF	1.84E-14	8
EGR1⟷PURA	Unknown	C2H2 ZF	8.00E-13	9
ZHX1⟷HINFP	Homeodomain	C2H2 ZF	2.93E-09	1
E2F4⟷HINFP	E2F	C2H2 ZF	1.18E-08	1
PURA⟷SP4	Unknown	C2H2 ZF	3.50E-06	1

Table S4: Mean and median information content for IR and non-IR filters for the three architectures: Basset-like, convolution and multi-head attention (CNN-MHA), and convolution with both recurrent network and multi-head attention (CNN-RNN-MHA).

Architecture	IR filters		Non-IR filters	
	Mean	Median	Mean	Median
Basset-like	4.12	4.21	4.26	4.26
CNN-MHA	4	4	4.4	4.38
CNN-RNN-MHA	3.9	3.85	4.42	4.41

Table S5: Mean and median information content for IR and non-IR filters with significant hits in the human TF database with q-value < 0.01 for the three architectures: Basset-like, CNN-MHA, and CNN-RNN-MHA.

Architecture	IR filters		Non-IR filters	
	Mean	Median	Mean	Median

Basset-like	4.34	4.21	4.23	4.09
CNN-MHA	4.43	4.42	4.95	4.9
CNN-RNN-MHA	4.47	4.48	4.75	4.92
