# Supplementary material for: Evidence for the role of transcription factors in the co-transcriptional regulation of intron retention
Source: Genome Biol. 2023 Mar 22;24:53. doi: 10.1186/s13059-023-02885-1 (PMC10031921; doi:10.1186/s13059-023-02885-1)
Supplement: Supplementary file 2 — Additional file 2. Review History. [file 13059_2023_2885_MOESM2_ESM.docx]

Review History

**First round of review**

**Reviewer 1**

**Are you able to assess all statistics in the manuscript, including the appropriateness of statistical tests used?** Yes, and I have assessed the statistics in my report.

**Comments to author:**

Summary:

This work investigates possible connections between transcription factors binding and intron retention. The authors first train a DL model to distinguish between DHS sites in introns that exhibit intron retention (IR), and DHS sites in other intergenic regions. They then analyzed the learned filters of their CNN by matching them to known motifs using Tomtom and checking for matching enrichment of ChiP-Seq peak calls of the associated TF in those motif locations. They then ran their recently developed SATORI algorithm to identify pairs of TF that seem to co-occur, reporting 400 such pairs.

The paper aims to address an important question that has been at the center of many RNA splicing research works, regarding the interplay between transcription and RNA processing and the mechanisms by which such crosstalk can occur. We definitely appreciated the amount of work that went into training the models presented here and performing the consequent analysis to shed light on this crosstalk. We also appreciated the detailed discussion section where the authors made sure to

We did have, however, several major concerns. To begin, all the methods and models employed in this work are not new and have been published by the associated groups before. The only difference is training those models on the positive/negative samples associated with IR presented here. Thus, the paper hinges on the ability to derive new biological insights. These, in turn, seemed limited by several key factors:

(1)
The methods are severely lacking and make it impossible to assess the reasonableness of the positive and negative sets used. How IR is defined is crucial in this work and the only thing reported here is that intron retention events were defined "using code from SpliceGrapher and iDiffIR". There is no reference to which RNA-seq datasets (which both tools require as input) were used in these definitions, which parameters and cut off were used, etc.

(2)
Given the above it is very hard to assess whether the IR happens in the same conditions in which TF binding is observed yes/no, and any context specificity.

(3)
Figure 1 could be made more clear by showing the data in a lengthed normalized manner. How much of Fig1 in intragenic is due to enhancers close to the TSS/in the first intron? Does IR versus non-IR introns show a bias in position in the gene? Are they closest to the transcription start site?

(4) Additional validation with ChIP
Every TF picked is very enriched. Can you do the same analysis with TFs your model does not think is influential and get no enrichment? What about the non-IR enriched motifs for SOX11, E2F4, and ZNF35...these should not be enriched. You can do the Table 1 and Figure 4 analysis on these?

Similarly why was this enrichment done for only the C2H2 ZF TF enrichments? Having a supplemental table with enrichment for all K562 TFs with ChIP could put these results in context. How noteworthy is the enrichments reported in Table 1? Is the model missing some biological signal in other TFs not captured well in the motif model?

How do TFs do vs. RBPs? Can you repeat analysis using ENCORE RBP eCLIP and get better results? Perhaps the motifs explain RBP binding better?

Interactions of motifs in IR. Of the 400+ interactions, 23 are in TRRUSTv2 (of 8324). There is probably ChIP for some of these TF interactions…can you look at if they're close together or not, like was done with motif proximity? Just check ChIP proximity.

It is unclear what is the value of all the fancy DL in the context given here: Couldn't you simply do this overlap and enrichment analysis using available ChIP-Seq?

No functional validations of the influence of TFs on IR. This relates also to the above comment about the value of the DL models (showing predictive capabilities). There is RNA-seq data available from ENCODE for some C2H2 ZF TFs in K562 cells like MAZ knockdown (https://www.encodeproject.org/experiments/ENCSR129WCZ/) and CRISPR KO of SP1 (https://www.encodeproject.org/experiments/ENCSR650QAK/) or SP2 (https://www.encodeproject.org/experiments/ENCSR715EDZ/). Based on the results here, one might expect to see alterations in intron retention levels upon depletion of these TFs. Similarly TF depletion for those associated with non-IR introns (e.g. E2F4 in K562 cells https://www.encodeproject.org/experiments/ENCSR874ZXG/) could also be examined. Are the TFs associated with non-IR events promoting efficient splicing (i.e. E2F4 depletion followed by RNA-seq would show increased levels of IR)? These functional results could greatly strengthen the manuscript and help elucidate mechanisms. Furthermore, the authors are probably aware that as it stands all the observations made here are just based on enrichment and associations. First, they could be some artifact of the way data is processed at the different stages (see also request for clarification on the pipeline/methods used), but even if these are "real" they can still be just that - non causal associations (e.g. via a shared mediator). Showing at least some support of connection (e.g. via the KD experiments) would help to support the significance of reported results.


Other comments:
—--------------------------
"More than 50% of IR have significant hits to C2H2 ZF TFs"...how many non-IR introns have this?

There is no ability to reproduce the methods and results presented in this manuscript in the current state of information and methods description.

**Reviewer 2**

**Are you able to assess all statistics in the manuscript, including the appropriateness of statistical tests used?** Yes, and I have assessed the statistics in my report.

**Comments to author:**

In this manuscript, Ullah et al. described a deep learning based method to discover transcription factors (TFs) associated with intron retention (IR) events. The deep learning model is first trained to distinguish between open chromatin regions in retained introns and non-retained introns, which achieves decent accuracy. The learned motifs of the model are further used to search for TFs associated with the IR events. The Zinc finger family of TFs is found highly enriched in IR events, and some of them are then supported by the following ChIP-seq analysis. Interactions of discovered TFs are further extracted from the model, in which significant overlaps are found in databases.

The manuscript is well written, methods are clearly described. The idea of discovering the roles of TFs in alternative splicing through deep learning is novel. Yet, in my opinion, improvements could be made to better characterize the authors' findings, especially more discussions on the discovered TFs and their regulatory mechanisms should be made. Accordingly, I have a couple of concerns that should be addressed.

1) The data are split into training, validation, and test sets for parameter tuning and model evaluation. Did the authors consider homologs in the process of data split? Is there an information leak if homologs are split into different sets, which can lead to overfitting?
2) The authors mentioned 23 IR motifs and 25 non-IR motifs have hits against multiple TFs. All the TFs matches are important information that should be listed in the supplementary materials.
3) The authors tried different choices of models and architectures, each with different discovered TFs in IR events. Even though the C2H2 ZF is always the dominant TF family, the percentages of overlaps of found TFs between different models should be reported and discussed.
4) The occupancy profiles across IR and non-IR events are shown for only two cases of TFs in Figure 4. To test the generality of the authors' interesting finding, the occupancy profiles for the other four TFs in Table 1 should be shown and discussed as well.
5) I am a bit disappointed that the authors didn't further validate the found TF interactions in IR events besides searching against the databases. Do the interacted TF pairs have correlations in terms of their occupancy profiles? Do the introns regulated by different numbers of TFs show different levels of retention?

Minor comments:
1) Some supplementary figures and tables are not referred in the main text.

**Authors Response**

I believe we addressed all the comments made by the reviewers. Reviewer 1 asked for functional validation of our computational predictions, and recommended the analysis of several knockdown experiments. We have done that, and the results support the conclusions we made in our original submission, and provide additional insights. Below is a detailed response.

We would like to thank the reviewers for their very insightful comments that have allowed us to make significant improvements to our manuscript.

For your convenience, here is a list of the major changes in the manuscript:
- Added an analysis of RNA-seq of knockdowns of several IR-associated TFs
- Added statistics on TF enrichment of additional TFs, including “negative controls”
- Performed an analysis of RBP binding motifs detected by the network.
- Additional validation of TF cooperativity using ChIP-seq data
- Minor additions in the introduction to reflect new research in deep learning in genomics
- During our revision we discovered several duplicate DHSs and some sequences that exhibited more than 80% sequence identity across the train and test sets (details below). Removing those sequences and retraining the classifiers yielded no change in classifier accuracy.
- We cleaned up the github repository and added additional code and to support reproducibility of our work.

Sections in the manuscript that were modified were highlighted in yellow.

Next, is our detailed response to reviewer comments.

*Reviewer #1: Summary:

This work investigates possible connections between transcription factors binding and intron retention. The authors first train a DL model to distinguish between DHS sites in introns that exhibit intron retention (IR), and DHS sites in other intergenic regions. They then analyzed the learned filters of their CNN by matching them to known motifs using Tomtom and checking for matching enrichment of ChiP-Seq peak calls of the associated TF in those motif locations. They then ran their recently developed SATORI algorithm to identify pairs of TF that seem to co-occur, reporting 400 such pairs.

The paper aims to address an important question that has been at the center of many RNA splicing research works, regarding the interplay between transcription and RNA processing and the mechanisms by which such crosstalk can occur. We definitely appreciated the amount of work that went into training the models presented here and performing the consequent analysis to shed light on this crosstalk. We also appreciated the detailed discussion section.

We did have, however, several major concerns. To begin, all the methods and models employed in this work are not new and have been published by the associated groups before. The only difference is training those models on the positive/negative samples associated with IR presented here. Thus, the paper hinges on the ability to derive new biological insights. These, in turn, seemed limited by several key factors:

(1)
The methods are severely lacking and make it impossible to assess the reasonableness of the positive and negative sets used. How IR is defined is crucial in this work and the only thing reported here is that intron retention events were defined "using code from SpliceGrapher and iDiffIR". There is no reference to which RNA-seq datasets (which both tools require as input) were used in these definitions, which parameters and cut off were used, etc.*

In order to not bias our analysis towards a particular cell line or tissue, we used intron retention events extracted from the Ensemble gene models. In the methods we have the following statement:
“Next, we extracted IR events from the Ensembl GRCh37 (hg19) reference annota- tions, utilizing code from SpliceGrapher [66] and iDiffIR [45].”
The script for extracting those events is now included in the project’s github repository.
We also added further clarification the use of annotated IR in the results section.

*(2)
Given the above it is very hard to assess whether the IR happens in the same conditions in which TF binding is observed yes/no, and any context specificity.*

That is true. It was not our intent to determine the conditions under which each TF affects IR – this work is merely a proof of concept to motivate future work on tissue- or cell-line-specific IR. This will require analysis of extensive RNA-seq data, which is our plan for future work.


*(3)
Figure 1 could be made more clear by showing the data in a lengthed normalized manner. How much of Fig1 in intragenic is due to enhancers close to the TSS/in the first intron? Does IR versus non-IR introns show a bias in position in the gene? Are they closest to the transcription start site?*

We decided to replace the original figure which used TF motif hits with peak information extracted from ChIP-seq experiments for a selection of TFs. Counts are now shown as number of peaks per MB, which should be well-normalized, and as suggested, removed from consideration the promoter and first intron/exon since they would have biased the results as observed by the reviewer.


*(4) Additional validation with ChIP
Every TF picked is very enriched. Can you do the same analysis with TFs your model does not think is influential and get no enrichment? What about the non-IR enriched motifs for SOX11, E2F4, and ZNF35...these should not be enriched. You can do the Table 1 and Figure 4 analysis on these?*

We added to our analysis a non-IR TF as well as TFs that were not detected as a match to any of the convolutional. All of these show the opposite trend when it comes to enrichment, i.e. are enriched in non-IR events.
Figure 4 was removed since we were not absolutely sure we normalized the coverage correctly (generating the figure is much more involved than the data in the table, which simply counts the percentage of events that overlap a ChIP-peak, and did not generate the expected result for negative controls).

*Similarly why was this enrichment done for only the C2H2 ZF TF enrichments? Having a supplemental table with enrichment for all K562 TFs with ChIP could put these results in context. How noteworthy is the enrichments reported in Table 1? Is the model missing some biological signal in other TFs not captured well in the motif model?*

We reported enrichment of additional TFs in our revision. Unfortunately, we didn’t have the time to compute TF-ChIP for all K562 TFs.

*How do TFs do vs. RBPs? Can you repeat analysis using ENCORE RBP eCLIP and get better results? Perhaps the motifs explain RBP binding better?*


Thanks for the suggestion. We added an analysis of RBP matches. Compared to TFs, there were very few RBPs that matched the IR/non-IR motifs (three for the IR motifs, and two for the non-IR motifs). This is reported in the Results section in the segment titled "The Zinc finger family of TFs are enriched in IR events". In view of the focus of the manuscript, we feel that analysis of eCLIP data is beyond the scope of this work.


*Interactions of motifs in IR. Of the 400+ interactions, 23 are in TRRUSTv2 (of 8324). There is probably ChIP for some of these TF interactions…can you look at if they're close together or not, like was done with motif proximity? Just check ChIP proximity.*

We have done so for two interactions. The following text was added to describe this experiment:
“As further support for predicted interactions, we looked at ChIP-seq data in K562 for the interactions EGR1-MAZ and EGR1-ZNF263 and evaluated the overlap between the peaks in intragenic regions. For the EGR1-MAZ interaction we found 21592 intragenic peaks for EGR1 and 16613 for MAZ. Out of those peaks, 9065 were within 150 bp of each other. Using Locus Overlap Analysis [49,50] to evaluate the significance of the overlap we obtained a p-value of 0. For the EGR1-ZNF263 interaction we found 21592 intragenic ChIP-seq peaks for EGR1 and 1619 for ZNF263. Out of those peaks, 715 were within a window of 150 bp of each other with a p-value of 1E-17.”

*It is unclear what is the value of all the fancy DL in the context given here: Couldn't you simply do this overlap and enrichment analysis using available ChIP-Seq?*

Without the deep learning results that showed that IR vs non-IR DHSs can be accurately predicted, we wouldn’t have had a good enough reason to do such an extensive screening. In future work we are planning to combine large-scale deep learning models trained on large scale TF-ChIP-seq into our modeling strategy, which will negate the need for such a screen and will give us the best of both worlds, enabling more reliable and accurate identification of TFs.

*No functional validations of the influence of TFs on IR. This relates also to the above comment about the value of the DL models (showing predictive capabilities). There is RNA-seq data available from ENCODE for some C2H2 ZF TFs in K562 cells like MAZ knockdown and CRISPR KO of SP1 or SP2. Based on the results here, one might expect to see alterations in intron retention levels upon depletion of these TFs. Similarly TF depletion for those associated with non-IR introns (e.g. E2F4 in K562 cells) could also be examined. Are the TFs associated with non-IR events promoting efficient splicing (i.e. E2F4 depletion followed by RNA-seq would show increased levels of IR)? These functional results could greatly strengthen the manuscript and help elucidate mechanisms. Furthermore, the authors are probably aware that as it stands all the observations made here are just based on enrichment and associations. First, they could be some artifact of the way data is processed at the different stages (see also request for clarification on the pipeline/methods used), but even if these are "real" they can still be just that - non causal associations (e.g. via a shared mediator). Showing at least some support of connection (e.g. via the KD experiments) would help to support the significance of reported results.*

Thank you for this suggestion.
We performed this analysis whose results are found in a new section “RNA-seq of TF Knockdowns suggests IR TFs function as splicing enhancers”. As we finished this analysis we found that E2F4 was included erroneously included in the non-IR motif hits. In all four cases we found increased levels of IR where most of the differential IR events lead to increased retention. We have also found that the motif of each TF is enriched in the exons flanking cases of differentially up-regulated IR events (Table 2 summarizes these results). All these suggest that these TFs function as splicing enhancers. We are currently working on doing a similar analysis for FOXK2, which is a non-IR TF. The results will be added in the next revision.

*Other comments:
—--------------------------
"More than 50% of IR have significant hits to C2H2 ZF TFs"...how many non-IR introns have this?*

As shown in Figure 3, 4/25 (around 12%) of non-IR motifs have hits to C2H2 ZF TFs.


*There is no ability to reproduce the methods and results presented in this manuscript in the current state of information and methods description.*

We added additional information in the Methods and added the code for extraction of IR events to the github repository.


*Reviewer #2: In this manuscript, Ullah et al. described a deep learning based method to discover transcription factors (TFs) associated with intron retention (IR) events. The deep learning model is first trained to distinguish between open chromatin regions in retained introns and non-retained introns, which achieves decent accuracy. The learned motifs of the model are further used to search for TFs associated with the IR events. The Zinc finger family of TFs is found highly enriched in IR events, and some of them are then supported by the following ChIP-seq analysis. Interactions of discovered TFs are further extracted from the model, in which significant overlaps are found in databases.

The manuscript is well written, methods are clearly described. The idea of discovering the roles of TFs in alternative splicing through deep learning is novel. Yet, in my opinion, improvements could be made to better characterize the authors' findings, especially more discussions on the discovered TFs and their regulatory mechanisms should be made. Accordingly, I have a couple of concerns that should be addressed.

1) The data are split into training, validation, and test sets for parameter tuning and model evaluation. Did the authors consider homologs in the process of data split? Is there an information leak if homologs are split into different sets, which can lead to overfitting?*

Since the DHS analysis focuses on intronic sequences we were not concerned about paralogs. However, using CDHIT, we detected 72 duplicate DHSs in the original Kelley et al. dataset. Some of these occurred across train and test set. This is out of a total of 7500 training examples. At a threshold of 80% sequence identity we also discovered 39 sequences whose similarity is above that threshold. We re-trained the classifier and found that the accuracy was unchanged. A note has been added in the Results section acknowledging this.

*2) The authors mentioned 23 IR motifs and 25 non-IR motifs have hits against multiple TFs. All the TFs matches are important information that should be listed in the supplementary materials.*

The information has been added in the project’s github repository under the results directory.

*3) The authors tried different choices of models and architectures, each with different discovered TFs in IR events. Even though the C2H2 ZF is always the dominant TF family, the percentages of overlaps of found TFs between different models should be reported and discussed.*

When comparing the TF hits for the IR motifs with an adjusted p-value of 0.01 or better for the three architectures, we find that 21 out of the 25 motif hits discovered by the purely convolutional architecture were common across the three deep learning architectures. 155 unique Bassett motifs out of 200 had significant hits using TomTom when searched against a library containing 200 motifs from the CNN-RNN-MHA architecture. For the CNN-MHA architecture we found 126 significant matches.

*4) The occupancy profiles across IR and non-IR events are shown for only two cases of TFs in Figure 4. To test the generality of the authors' interesting finding, the occupancy profiles for the other four TFs in Table 1 should be shown and discussed as well.*

We removed the occupancy profiles, since we were not absolutely sure that they were normalized correctly. We added results for additional TFs in Table 1 for a non-IR TF and other TFs that were not detected by the model as “negative controls”.

*5) I am a bit disappointed that the authors didn't further validate the found TF interactions in IR events besides searching against the databases. Do the interacted TF pairs have correlations in terms of their occupancy profiles? Do the introns regulated by different numbers of TFs show different levels of retention?*

We have done so for two interactions. The following text was added to describe this experiment:
“As further support for predicted interactions, we looked at ChIP-seq data in K562 for the interactions EGR1-MAZ and EGR1-ZNF263 and evaluated the overlap between the peaks in intragenic regions. For the EGR1-MAZ interaction we found 21592 intragenic peaks for EGR1 and 16613 for MAZ. Out of those peaks, 9065 were within 150 bp of each other. Using Locus Overlap Analysis [49,50] to evaluate the significance of the overlap we obtained a p-value of 0. For the EGR1-ZNF263 interaction we found 21592 intragenic ChIP-seq peaks for EGR1 and 1619 for ZNF263. Out of those peaks, 715 were within a window of 150 bp of each other with a p-value of 1E-17.”
Our analysis did not consider tissue- or cell-line-specific IR, so questions regarding IR levels were not addressed.

*Minor comments:
1) Some supplementary figures and tables are not referred in the main text.*

Fixed!

**Second round of review**

**Reviewer 2**

The authors have properly addressed most of the concerns I raised ealier. However, the following minor revison is needed before acceptance.

1. The response to question 3 of reviewer 2 shoud be included in the main text. It is worth showing to the readers that the model is robust to different variants and the found motifs are trustworthy.
